# Evaluation of thyroglobulin autoantibodies in dogs at the time of diagnosis of hypoadrenocorticism and during treatment

**DOI:** 10.1002/vetr.70622

**Published:** 2026-04-28

**Authors:** Christin Emming, Sina Strey, Ina Leiter, Reinhard Mischke, Holger Volk, Marion Schmicke, Johanna Rieder

**Affiliations:** ^1^ Department of Small Animal Medicine and Surgery University of Veterinary Medicine Hannover Hannover Germany; ^2^ Department of Veterinary Endocrinology and Laboratory Diagnostics University of Veterinary Medicine Hannover Hannover Germany

**Keywords:** autoimmune, dog, hypoadrenocorticism, polyendocrine syndrome, thyroiditis

## Abstract

**Background:**

Autoimmune thyroiditis (AIT) may occur more frequently in dogs with hypoadrenocorticism (HA) than previously recognised. The objective of this study was to determine the presence of thyroglobulin autoantibodies (TgAAs) in dogs with HA.

**Methods:**

Dogs were included if HA was confirmed by an adrenocorticotropic hormone stimulation test conducted between 2019 and 2023. Serum total thyroxine, thyrotropin and TgAA were analysed from samples taken before and during HA treatment.

**Results:**

Thirty‐seven dogs were enrolled, with nine dogs showing positive TgAA titres at enrolment. Following HA treatment, TgAA became undetectable in six dogs; two showed persistent positive TgAA results and one was lost to follow‐up. Additionally, two dogs that were initially TgAA negative developed hypothyroidism during the follow‐up period. The overall prevalence of concurrent HA and hypothyroidism after initiation of hormone replacement therapy was three of 30 among the dogs for which follow‐up data were available.

**Limitations:**

The retrospective nature of the study, small sample size and incomplete follow‐up data for some patients limit the conclusions that can be drawn.

**Conclusion:**

Our findings demonstrate the concurrent occurrence of two different autoimmune diseases, AIT and HA, and show that the presence of TgAA can completely resolve with cortisol replacement therapy. Therefore, it is recommended to reassess thyroid function after initiating HA treatment to avoid unnecessary lifelong thyroxine supplementation.

## INTRODUCTION

Hypoadrenocorticism (HA) is a rare endocrine disease. However, a higher incidence is reported in dogs than in other species, with prevalences up to 100 times higher than those observed in people.^1^, ^2^ The most common cause of primary HA in both species is the autoimmune‐related destruction of the adrenal cortex, with previous histopathological studies showing lymphoplasmacytic inflammation in the adrenal cortex.^3^, ^4^ Most autoimmune diseases are characterised by the presence of autoantibodies targeting host tissues, resulting in tissue destruction caused by autoreactive lymphocytes.^5^ Hypothyroidism in dogs can be the result of an autoimmune thyroiditis (AIT) with infiltration of lymphocytes in the thyroid glands and subsequent tissue damage, which results in functional failure.^6^, ^7^ Canine hypothyroidism shares many similarities with human Hashimoto's thyroiditis.^8^ Circulating thyroglobulin autoantibodies (TgAAs), which target thyroglobulin, the major protein of the thyroid gland, have been detected in 60%–80% of people and approximately 50% of dogs with hypothyroidism and are regarded as a specific indicator of AIT.^9^, ^10^, ^11^, ^12^

The progression of AIT is insidious and consists of four distinct stages.^6^ The first, subclinical (or silent) thyroiditis, involves lymphocytic infiltrates in the thyroid gland with detectable TgAA, while total thyroxine (TT4) and thyrotropin (TSH) levels remain normal.^6^, ^13^, ^14^ The subsequent stage, antibody‐positive subclinical hypothyroidism, is marked by pathological changes in 60%–70% of the thyroid tissue, causing a compensatory TSH increase, with TgAA still present.^15^ As a result of compensatory TSH secretion, TT4 concentrations remain within the normal range during the subclinical stage. As the disease progresses, TT4 concentration decreases, and clinical hypothyroidism becomes apparent in the third stage, known as antibody‐positive overt hypothyroidism, where thyroid follicles are almost completely destroyed.^6^ This stage is characterised by reduced TT4, increased TSH and detectable TgAA. The final stage, non‐inflammatory atrophic hypothyroidism, is characterised by an atrophic histopathological appearance and the disappearance of TgAA.^13^

The measurement of TT4 provides excellent diagnostic sensitivity; however, its specificity is compromised due to the influence of exogenous factors such as medications or other non‐thyroidal diseases.^15^ Furthermore, breed‐specific reference intervals (RIs) should be considered. On the other hand, TSH screening offers high specificity but limited sensitivity, as approximately one‐quarter of hypothyroid dogs may have TSH levels within the RI.^16^ According to recent guidelines, a definitive diagnosis of hypothyroidism is characterised by TT4 and free T4 (fT4) concentrations below the RI, along with a TSH concentration above the RI.^17^ However, up to 40% of hypothyroid dogs may show low TT4 and low fT4 but normal TSH concentrations, a pattern that can also occur in non‐thyroidal illness and may complicate diagnosis.^18^ In such scenarios, when clinical suspicion remains high, further diagnostic testing (e.g., TSH or Thyrotropin‐Releasing Hormone (TRH) stimulation testing) is warranted.^17^, ^19^ The percentage measurement of TgAA binding is widely used and is regarded as highly specific, with positive results typically indicating underlying AIT and only rarely being associated with non‐thyroidal illnesses.^14^, ^16^ A recent study on hypothyroidism in Eurasians underscores the value of TgAA as a predictive marker for the development of hypothyroidism.^13^ In addition to TgAA, assays for the detection of autoantibodies targeting TT4, triiodothyronine (T3) and thyroid peroxidase (TPO) have been described in the literature, although they are not always commercially available.^20^

The dysfunction and involvement of two or more immune‐mediated endocrinological diseases is defined as autoimmune polyendocrine syndrome (APS).^2^, ^21^, ^22^ This is well described in human medicine. According to previous studies, at least half of human patients who suffer from HA have autoimmune comorbidity.^23^, ^24^, ^25^ The combination of HA and AIT, which is the most commonly observed, is classified as APS‐II, also known as Schmidt syndrome.^26^, ^27^ In veterinary medicine, autoimmune comorbidities have rarely been reported in individual case reports and a few retrospective studies.^21^, ^22^, ^28^, ^29^ The overall prevalence of APS in dogs was 4.9% (11/225 dogs) according to one retrospective study.^28^ Blois et al. reported that the combination of HA and AIT being the second most common constellation, with an incidence of 22.9% (8/35 dogs).^22^

To the authors’ knowledge, only limited data are available regarding the presence and progression of TgAA during the treatment course in dogs with HA.^30^ The objectives of the current study were to ascertain the prevalence of AIT based on TgAA, TT4 and TSH testing in dogs at the time of HA diagnosis and during treatment. We hypothesised that positive TgAA titres might be more prevalent in dogs with HA than previously expected.

## MATERIALS AND METHODS

### Study cohort

Medical records of client‐owned dogs presented to the Department of Small Animal Medicine and Surgery at the University of Veterinary Medicine Hannover between 2019 and 2023 were retrospectively screened and enrolled in the study. A subset of the baseline data was obtained from a pilot study.^31^ The diagnostic workup included a complete blood count, biochemical analysis and adrenocorticotropic hormone (ACTH) stimulation test. Except for two dogs, all dogs underwent detailed abdominal ultrasound examination (35/37 patients). Dogs were included if their 1‐hour post‐ACTH serum cortisol concentration was below 55.2 nmol/L and if their clinical presentation was consistent with HA. Dogs were excluded if they were pretreated with glucocorticoids (oral, parenteral, topical) in the 6 weeks before the ACTH stimulation test or if they were undergoing therapy controls as part of trilostane therapy. Furthermore, none of the dogs had received gestagen hormone treatment with potential glucocorticoid activity. Follow‐up data were available for all dogs except for seven and were collected during routine monitoring of HA treatment.

### Analytical testing

For the ACTH stimulation test, blood samples were taken before and 1 hour after administering an intravenous dose of 5 µg/kg synthetic ACTH (Cosacthen, Dechra Veterinary Products Deutschland, or Synacthen, Alfasigma). Blood samples were collected into serum tubes and centrifuged after clotting. The serum was transferred into plastic tubes and promptly transported to the clinical endocrinology laboratory at the University of Veterinary Medicine Hannover. Cortisol measurements were conducted promptly, and the serum was subsequently stored at ‒20°C until thyroid parameters were analysed. Serum cortisol concentrations were determined using the chemiluminescent enzyme immunoassay (IMMULITE 2000 Cortisol Assay, VCOR) on the IMMULITE 2000 analyser (Siemens Healthineers). A pooled serum sample from randomly selected healthy dogs was tested 10 times to determine the intra‐assay coefficient of variation (CV), which was 7.8% (medium concentration).

For the measurement of serum TT4 and TSH, a two‐site chemiluminescent immunometric assay based on the IMMULITE 1000 analyser was used (IMMULITE assays, canine total T4 and canine TSH, Siemens Healthineers). The detection limit of the TT4 test was 1.5 nmol/L, and that of the TSH assay was 0.01 ng/mL. The intra‐assay CV was determined by analysing two pooled sera (low and high concentrations) 10 times in one test routine, while the inter‐assay CV was calculated by measuring the pooled sera on 10 consecutive days. The intra‐assay CVs for TT4 and TSH were 5.8% and 7.2%, respectively. Meanwhile, the inter‐assay CVs for TT4 and TSH were 8.2% and 8.5%, respectively. RI used for TT4 was 14.2–55.5 nmol/L, and for TSH, RI was less than 5.05 mU/L. In dogs diagnosed in 2023, the concentrations of TT4 and TSH were measured using the IMMULITE 2000 analyser, and the RI for TT4 changed to 28.1–55.5 nmol/L.

The detection of TgAA was performed using the canine TgAA enzyme immunoassay (VT10, TgAA EIA kit, Oxford Laboratories). The RI for TgAA was less than 10%, with values between 10% and 25% considered borderline. All results in this study were confirmed by analysing TgAA a second time using the TgAA immunoassay with non‐specific binding (VT12, TgAA EIA kit, Oxford Laboratories). The intra‐assay CVs were 3.2% and 11.7%, respectively, as determined in‐house using pooled sera from randomly selected dogs, measured 10 times.

### Clinical outcome

Follow‐up after the start of HA treatment was conducted for most of the included dogs. Follow‐up data for all but one of the dogs with elevated TgAA titres at enrolment were available for review. The frequency of follow‐up visits varied among patients. Convenience sampling was used to collect data, and surplus serum samples obtained during these rechecks were analysed for thyroid parameters

### Thyroid status categorisation using TgAA, TT4, and TSH measurements

The classification of enrolled dogs into different groups based on their thyroid results at the time of HA diagnosis and during follow‐up was guided by criteria from previous studies.^6^, ^13^ Initially, the dogs were divided into those that were TgAA‐positive (group A) and those that were TgAA‐negative (group B). Subsequently, the dogs were further categorised into the following groups:

1. Normal TT4 and TSH
2. Normal TT4 and increased TSH
3. Hypothyroidism (decreased TT4 and increased TSH)
4. Potential non‐thyroidal illness (decreased TT4 and non‐elevated TSH)

### Statistical analysis

Statistical analysis was performed using GraphPad Prism version 10 (GraphPad Software). The normality of the collected data was assessed using the Shapiro‒Wilk tests. Prevalences were presented as absolute numbers and percentages, including 95% confidence intervals (CIs), which were calculated using the Wald method. In the descriptive statistics, normally distributed data are presented as mean (± standard deviation [SD]), and non‐normally distributed data are presented as median (interquartile range [IQR]). Given the different TT4 reference ranges, *Z*‐standardisation was conducted to facilitate statistical analysis. The comparisons between TgAA‐positive and TgAA‐negative dogs for age, weight, hospitalisation status and initial TT4 and TSH concentrations were performed using the Mann‒Whitney test for non‐parametric data and the unpaired *t*‐test for parametric data. Changes in TgAA, TT4 and TSH concentrations in TgAA‐positive dogs between the time of HA diagnosis and follow‐up were analysed using the Wilcoxon test for non‐parametric data and the paired *t*‐test for parametric data. The results were considered statistically significant at a *p*‐value of less than 0.05.

## RESULTS

### Study population

Thirty‐seven dogs with confirmed HA were included in the study. Of these dogs, 20 were male (14 neutered) and 17 were female (10 neutered), with a median age of 4.96 years (IQR: 3.1–7.2 years). The mean bodyweight at the time of HA diagnosis was 16.7 kg (SD: ±9.5 kg). The cohort consisted of 12 crossbreed and 25 purebred dogs. Among the purebred dogs, there were three Jack Russell Terriers, two Labrador Retrievers and two Portuguese Water Dogs. The remaining breeds were each represented by one dog and included: Pug, Bodeguero Terrier, Airedale Terrier, Magyar Vizsla, Boston Terrier, Bolonka, German Shorthaired Pointer, Labradoodle, Papillon, Australian Terrier, Australian Shepherd dog, Samoyed, Cocker Spaniel, Miniature Wire‐Haired Dachshund, Poodle, Chihuahua, Nova Scotia Duck Tolling Retriever and French Bulldog.

At the initial presentation, 25 dogs (68%; 95% CI: 51%–80%) were presented with hyperkalaemia and hyponatraemia, while one dog exhibited only hyperkalaemia. Eukalaemia and hyponatraemia were observed in five dogs, and six dogs had unremarkable electrolyte levels. Initially, seven dogs were managed as outpatients, while 30 dogs required hospitalisation with a median stay of 5 days (IQR: 3–6 days).

All dogs initially received prednisolone at a median starting dose of 0.5 mg/kg/day intravenously or orally (IQR: 0.2–0.5 mg/kg every 12‒24 hours), based on the patient's individual needs. In addition to glucocorticoid therapy, most dogs initially received mineralocorticoid therapy, either with fludrocortisone (Astonin H 0.1 mg tablets, Merck; 0.01 mg/kg orally every 12 hours) or deoxycorticosterone pivalate (Zycortal, Dechra Veterinary Products Deutschland; median: 1.8 mg/kg; IQR: 1.5–2.1 mg/kg subcutaneously every 28 days). Six dogs with eukalaemic and eunatraemic HA were exclusively treated with prednisolone for long‐term therapy. Thirty dogs were available for ongoing monitoring, while seven dogs were lost to follow‐up. Dosage adjustments of glucocorticoid and mineralocorticoid therapy were individualised based on vital signs, laboratory parameters and potential adverse effects. In one dog, prednisolone treatment was discontinued, and only fludrocortisone (0.01 mg/kg/day) was administered. The mean prednisolone dose for the remaining 29 dogs during long‐term therapy was 0.14 mg/kg/day (SD: ±0.06 mg/kg/day).

### Evaluation of serum TT4, TSH and TgAA binding at time of diagnosis of HA

In this study population, nine of 37 dogs (24%; 95% CI: 13%–40%) had a TT4 concentration below the RI. TSH level was above the RI in eight of 37 dogs (22%; 95% CI: 11%–37%) with a median concentration of 2.92 mU/L (IQR: 1.1–4.8 mU/L). The overall prevalence of elevated TgAA binding (group A) was nine of 37 (24%; 95% CI: 13%–40%), as illustrated in Figure 1. The thyroid parameters obtained in group A are summarised in Table 1. The median TgAA binding in group A was 47% (IQR: 15.4%–119.4%), with four dogs showing borderline TgAA titres. There was no significant difference in TT4 and TSH concentrations between groups A and B (*p* = 0.256 and 0.118, respectively), as demonstrated in Figure 2. Age, sex, weight and hospitalisation time did not significantly differ between groups A and B (age, *p* = 0.893; weight, *p* = 0.964; hospitalisation, *p* = 0.529).

**FIGURE 1:**
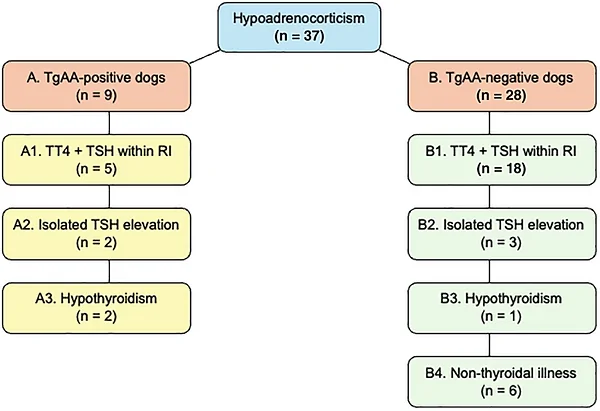
Flow chart showing thyroid‐based patient grouping. The study cohort consisted of 37 dogs with confirmed hypoadrenocorticism. According to the presence of thyroglobulin autoantibodies (TgAAs), dogs were divided into group A (TgAA positive) or group B (TgAA negative). Subgroups were defined based on thyroid parameters: (1) total thyroxine (TT4) and thyrotropin (TSH) within the reference intervals (RIs); (2) isolated TSH elevation with TT4 within the RI; (3) hypothyroidism (decreased TT4 and increased TSH); and (4) potential non‐thyroidal illness (decreased TT4 and normal TSH).

**TABLE 1:** Serum concentrations of total thyroxine (TT4), thyrotropin (TSH) and thyroglobulin autoantibodies (TgAA) in dogs that were initially positive for TgAA (group A) at baseline and during hypoadrenocorticism (HA) treatment

Hormones		Dog 1	Dog 2	Dog 3	Dog 4	Dog 5	Dog 6	Dog 7	Dog 8	Dog 9	Hormone status (%)
Total T4 (nmol/L)	Initial	15.6	0.64	22.2	0.64	32.4	36.3	44.0	49.5	26.9	Below RI: 33 Within RI: 67
	Recheck	#27.7	*19.2	×	#9.7	#30.4	#35.1	#15.6	#47.5	#4.68	Within RI: 56 Above RI: 44
TSH (mU/L) RI < 5.05	Initial	1.0	40.36	2.92	59.42	3.37	7.64	1.8	6.49	1.75	Within RI: 56 Above RI: 44
	Recheck	0.35	4.2	×	0.35	0.35	1.91	0.35	6.85	1.46	Within RI: 87.5 Above RI: 12.5
TgAA (%) RI < 10%	Initial	17.04	427.34	87.34	151.4	81.95	16.9	12.6	46.9	13.8	Above RI: 100
	Recheck	<10	<10	×	32.4	<10	<10	<10	13.2	<10	Below RI: 75 Above RI: 25
Recheck interval (days)		1409	230	×	145	526	261	422	274	44	

*Note*: The proportion of cases with TT4, TSH and TgAA levels below, within or above the respective reference interval (RI) at each time point is shown in the last column. The last row indicates the time interval (days) between HA diagnosis and the individual recheck. Dog 3 was lost to follow‐up, and no additional data were available. TT4 RI 14.2–55.5 nmol/L (IMMULITE 1000) is marked as ‘*’, and 2023 cases (IMMULITE 2000): 28.1–55.5 nmol/L are marked as ‘#’.

**FIGURE 2:**
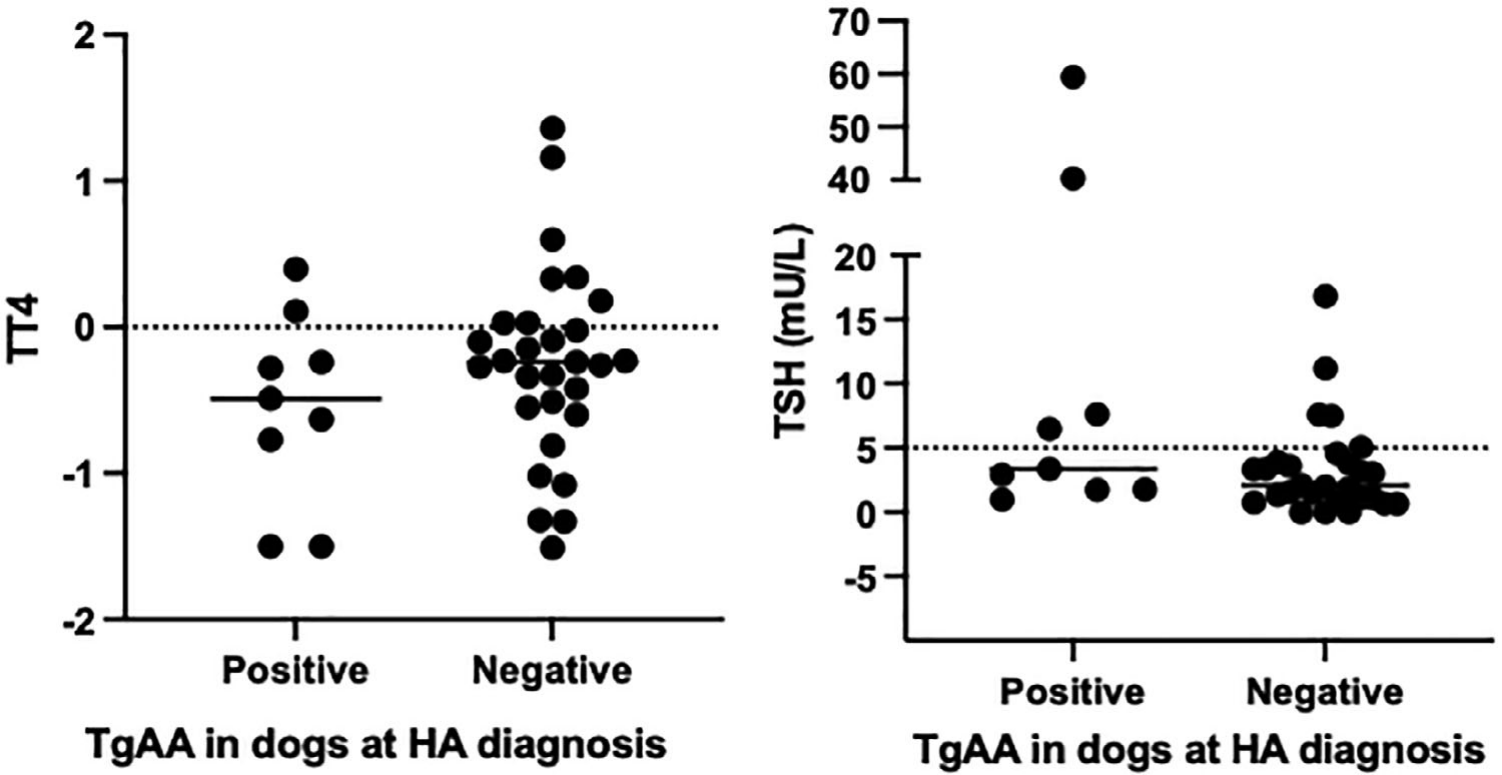
Comparison between dogs with positive (group A) or negative (group B) thyroglobulin autoantibodies (TgAAs) concentrations regarding total thyroxine (TT4) and thyrotropin (TSH) concentrations at the time of hypoadrenocorticism (HA) diagnosis. There was no significant difference between groups A and B in TT4 (*p* = 0.256) or TSH concentrations (*p* = 0.118). *Z*‐standardisation was applied to normalise differences between TT4 reference ranges. The dotted lines represent the upper limits of the reference interval.

### Thyroid parameter monitoring during HA therapy in dogs initially positive for TgAA

Eight of the nine dogs underwent routine follow‐up examinations for HA and thyroid status, and the data are presented in Table 1. At recheck, the mean prednisolone dose was 0.17 mg/kg/day (SD: ±0.06 mg/kg/day). One dog (dog 8) received only fludrocortisone (0.01 mg/kg/day), and prednisolone was omitted. Both TgAA titres and TSH concentrations decreased significantly between the initial and follow‐up examinations (*p* = 0.008 and 0.023, respectively). In contrast, TT4 values did not change significantly between the two time points (*p* = 0.373). All data on thyroid hormone changes are illustrated in Figure 3. TgAA titres were no longer detectable in six of eight dogs (75%; 95% CI: 40%–94%), while one dog (dog 8) had TgAA binding in the questionable range. The final dog (dog 4) consistently showed a threefold increase in TgAA (Table 1). In this dog, the TSH level was not detectable in the follow‐up examination, and the TT4 concentration increased during HA therapy but remained just below the RI. This dog also presented clinical signs consistent with hypothyroidism, leading to the initiation of levothyroxine treatment (Forthyron, Dechra Veterinary Products Deutschland; 10 µg/kg every 12 hours).

**FIGURE 3:**
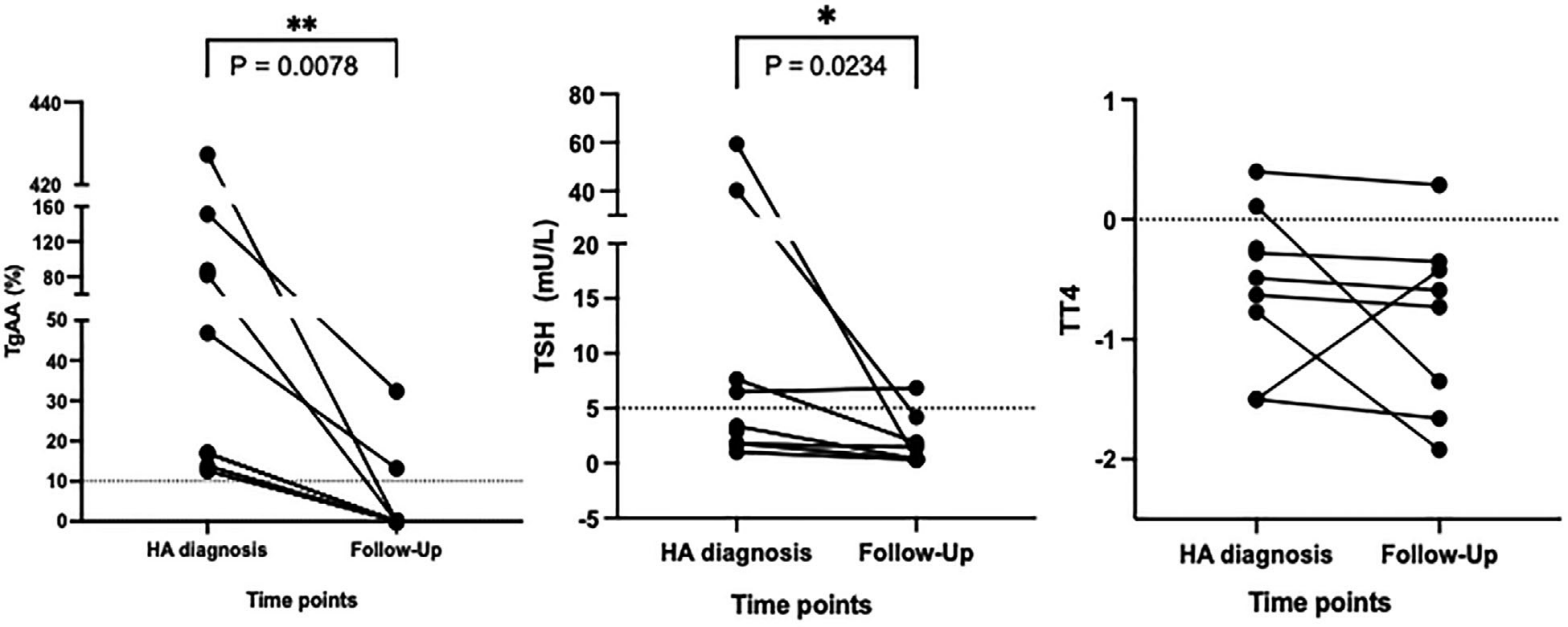
Changes in serum thyroglobulin autoantibodies (TgAAs), thyrotropin (TSH) and total thyroxine (TT4) concentrations in eight dogs with initially positive TgAA (group A) at the time of hypoadrenocorticism (HA) diagnosis and during follow‐up examinations after HA treatment. TgAA (*p* = 0.0078) and TSH (*p* = 0.0234) concentrations decreased significantly during HA therapy. TT4 concentrations did not differ significantly between time points (*p* = 0.3734). The significance levels are indicated as follows: ^*^ *p* < 0.05; ^**^ *p* < 0.01; ^***^ *p* < 0.001. The dotted lines represent the upper limit of the reference interval.

### Thyroid parameter monitoring during HA therapy in TgAA‐negative dogs

Twenty‐two dogs from group B were regularly monitored during HA therapy. As part of the HA controls, thyroid parameters were re‐evaluated in seven dogs due to clinical suspicion of hypothyroidism. In two of seven dogs, TT4 concentrations were reduced and contemporaneously increased TSH concentrations were confirmed. One of these dogs tested negative for TgAA, while TgAA was not measured in the other dog. In the remaining 15 dogs, thyroid parameters were not assessed because their clinical condition was unremarkable and there was no evidence of hypothyroidism.

### Overall prevalence of concurrent HA and hypothyroidism

At the time of HA diagnosis, two dogs from group A and one dog from group B were hypothyroid, resulting in a prevalence of concurrent HA and hypothyroidism of three of 37 (8%; 95% CI: 2%–22%). One of the TgAA‐positive dogs (dog 2) showed complete recovery from AIT, while another (dog 4) remained hypothyroid despite a decrease in TSH concentration. The dog from group B that was initially hypothyroid did not undergo follow‐up thyroid testing, but its clinical appearance was not consistent with hypothyroidism. During the course of the disease, two additional dogs from group B developed hypothyroidism. Consequently, the final overall prevalence of concurrent HA and hypothyroidism after hormone replacement therapy was three out of 30 dogs for which follow‐up data were available (10%; 95% CI: 3%–26%), including one TgAA‐positive (dog 4) and two TgAA‐negative dogs.

## DISCUSSION

The current study presents data on serum TgAA, TT4 and TSH concentrations in 37 dogs with HA before initiation of glucocorticoid and, in most cases, mineralocorticoid replacement therapy, as well as follow‐up data from a subset of dogs. In nearly one‐quarter of dogs with an HA diagnosis, TgAA serum levels were increased. Furthermore, TgAA and TSH serum levels normalised in most dogs following glucocorticoid therapy. Based on these results, TgAA and thyroid parameters in dogs recently diagnosed with HA should be interpreted with caution to avoid misdiagnosis of hypothyroidism.

In human medicine, patients suffering from HA have an increased risk of developing a second autoimmune disease. The most common additional disease is AIT, which occurs in approximately 50% of females and 25% of males.^25^ Interestingly, the female predisposition could not be confirmed in our canine study population. While numerous canine case reports suggest a female predilection,^21^, ^26^, ^32^, ^33^ a previous retrospective study also failed to establish a sex predisposition in dogs with HA and AIT.^22^ Furthermore, our study did not identify other predictors that are purportedly more strongly associated with TgAA positivity (e.g., age, weight, hospitalisation time and electrolyte changes).

In general, it is presumed that the prevalence of multiple autoimmune diseases in veterinary medicine is underestimated.^2^ A study conducted in the USA regarding autoimmune diseases in Italian Greyhounds reported an overall incidence of 9%. More than half of the dogs developed more than one autoimmune disease during their lifetime, such as HA and immune‐mediated haemolytic anaemia (IMHA), thyroiditis and IMHA, or meningitis and IMHA.^34^ A recently published study reported a prevalence of hypothyroidism in dogs with HA of 8% (5/66 dogs). In most cases, hypothyroidism was diagnosed after the diagnosis of HA, whereas in one dog, it was diagnosed concurrently with HA. Detectable TgAA was found in only one dog in which hypothyroidism was ultimately confirmed; however, TgAA levels were not evaluated in all dogs.^30^

In the present study, nine dogs tested positive for TgAA, indicating the presence of AIT at the time of HA diagnosis. Two of these dogs (dogs 2 and 4, Table 1) exhibited markedly elevated TgAA levels, along with TT4 and TSH concentrations significantly deviating from the RI, suggesting a diagnosis of hypothyroidism. All dogs received glucocorticoid replacement therapy for the treatment of adrenal insufficiency; however, no thyroid hormone supplementation was initiated in any of them. All initially TgAA‐positive dogs, except for one, underwent thyroid hormone reassessment during follow‐up at our veterinary clinic. Six out of eight dogs tested completely negative for TgAA within a few weeks. Dog 8 had a persistently elevated TSH concentration and a borderline TgAA titre, along with a normal TT4 concentration, indicating subclinical hypothyroidism (Table 1). In dog 4, a recheck examination was performed 145 days post‐diagnosis, showing normalisation of the TSH concentration with persistently positive TgAA and an abnormal TT4 concentration (Table 1). This dog was receiving physiological replacement therapy with prednisolone at a dosage of 0.11 mg/kg once daily. A second thyroid assessment was performed 230 days after diagnosis (data not shown). At that time, the dog was still receiving prednisolone at the same dosage. The TSH concentration remained within the RI, while TgAA remained increased and TT4 was decreased. The dog also showed clinical signs consistent with hypothyroidism (lethargy, weight gain and dull coat). Following initiation of levothyroxine therapy, the dog showed a favourable response. As previously mentioned, dog 2 initially showed laboratory diagnostic changes consistent with TgAA‐positive hypothyroidism, but the follow‐up examination confirmed a complete recovery of all thyroid parameters (Table 1). Interestingly, two dogs that initially tested negative for TgAA later developed hypothyroidism. This suggests that the prevalence of concurrent HA and hypothyroidism may be higher than previously described in dogs, although it remains significantly lower than what is reported in people.^23^ However, the small sample size due to the low prevalence of HA affects the robustness of the findings and limits the study's overall validity. Our results show that glucocorticoid replacement therapy led to remission of AIT in most cases, although it failed in some instances. Physiological and pharmacological doses of glucocorticoids exert a negative feedback mechanism on TSH levels by acting on the pituitary gland, but they do not directly affect TT4.^35^ This effect is absent in adrenal insufficiency and, consequently, the TSH concentration may be elevated at the time of HA diagnosis.^36^, ^37^ This phenomenon would explain the significant steroid‐responsive reduction in TSH levels in our study population, but an impact on autoimmunity by cortisol replacement therapy seems doubtful. Reusch et al. reported that 11 out of 30 dogs with HA exhibited elevated TSH concentrations at the time of diagnosis, which normalised quickly after the start of HA therapy.^38^ However, none of the dogs with TSH elevation showed a concomitant decrease in TT4 concentrations. This contrasts with our data, where two TgAA‐positive dogs showed both a decrease in TT4 level and an increase in TSH level, and one dog with TgAA‐negative hypothyroidism was also observed. In human medicine, only a few reports exist regarding normalisation of thyroid function under cortisol therapy in patients with HA.^35^, ^39^, ^40^

There are two possible explanations for the regression of the thyroid function during glucocorticoid replacement therapy. The first hypothesis is that prednisolone therapy in this specific formulation, despite the low dose, exerted an immunomodulatory effect in the cohort, resulting in resolution of autoimmunity in most animals. Recent studies report cytokine shifting from a pro‐inflammatory to an anti‐inflammatory state due to physiological steroid levels, highlighting an immunomodulatory effect.^41^ In our study cohort, the mean prednisolone dose in dogs initially positive for TgAA was 0.17 mg/kg/day, which corresponds to a physiological cortisol replacement dose and is consistent with this hypothesis.

The second possible explanation could be spontaneous remission of the thyroiditis and normalisation of TgAA, which has been reported in dogs without HA. A recent study by Egbert et al. assessed thyroid parameters in dogs with subclinical thyroiditis and found that 14% of initially TgAA‐positive dogs became TgAA‐negative without any treatment, and they remained euthyroid.^10^ Additionally, another study examined the presence of TgAA in hypothyroid dogs (*n* = 118) and in euthyroid dogs with non‐thyroidal diseases (*n* = 2496). In the latter group, TgAA was present in 6.3% of the cases (*n* = 156). In 14.1% of the cases, TgAA binding decreased, while 22.5% of the dogs eventually developed overt hypothyroidism.^13^, ^42^

A study by Boag et al. tested different antibodies in dogs with HA, and antibodies to the cytochrome P450 side‐chain cleavage enzyme (P450scc) appeared promising, as 51 out of 213 (24%) dogs with HA tested positive for P450scc antibodies.^1^ A follow‐up study analysed the presence of P450scc antibodies in 32 dogs over the long term, using repeat blood samples to observe the dynamics of the autoimmune response. Interestingly, five dogs remained antibody positive, in three dogs the antibody status became negative, one dog was initially negative and tested positive after 18 months, and 23 dogs remained negative.^43^ This indicates that autoantibodies can persist, disappear or develop newly over the course of the disease. The persistence of autoantibodies may reflect ongoing antigen stimulation and inflammation; therefore, retesting for autoantibodies in dogs with endocrine diseases of autoimmune aetiology is recommended.

Diagnosing hypothyroidism in dogs is generally challenging, as both individual thyroid parameters and the combination of laboratory results can lead to misdiagnosis. As mentioned earlier, TSH concentration can be temporarily elevated in untreated dogs with HA.^38^ Additionally, acute illness can affect thyroid hormone levels, sometimes resulting in an elevated TSH concentration during the recovery period; however, a recent study did not find concurrent low TT4 and elevated TSH concentrations.^18^

TgAA measurement is a well‐established marker of AIT, and a positive TgAA result supports the diagnosis of hypothyroidism when abnormal TT4 and TSH values are present.^8^, ^10^, ^13^, ^16^ Additionally, detectable TgAA can also predict the potential development of hypothyroidism.^13^ For this reason, TgAA titres are frequently used to guide breeding decisions.^10^ The prevalence of autoantibodies against TT4 and T3 is typically lower compared to TgAA, and they are usually detected alongside positive TgAA results.^5^, ^13^, ^44^ Thus, these autoantibodies are measured less frequently in veterinary medicine. Nevertheless, circulating autoantibodies against TT4 can cross‐react with the TT4 assay, causing falsely elevated TT4 results. As such, the detection of TT4 autoantibodies can still be a useful diagnostic tool.^17^

This study highlights that TgAA can be detected in a notable proportion of dogs with HA. Despite several similarities to the study by Sieber‐Ruckstuhl et al., our findings contrast with their data.^30^ In both studies, the same assay (ELISA; Oxford Laboratories) was used for TgAA measurement. However, in the present study, analyses were performed in‐house, whereas in the study by Sieber‐Ruckstuhl et al. measurements were conducted at Michigan State University.^30^ Although the number of dogs at the time of HA diagnosis was similar in both studies, the descriptive statistics reported by Sieber‐Ruckstuhl et al. were based on all dogs included over the entire disease course (*n* = 66).^30^ When comparing their full cohort with our study population, age and median bodyweight appeared broadly comparable. Their study included 62% female dogs, whereas 46% of the dogs in our cohort were female. Both studies comprised a wide range of purebred and mixed‐breed dogs, with overlap in several breeds (e.g., Jack Russell Terrier, Labrador Retriever, Poodle and Labradoodle). In contrast to the findings of Sieber‐Ruckstuhl et al.,^30^ in which only one dog developed increased TgAA concentrations and hypothyroidism during the course of HA, we identified increased TgAA concentrations in nine dogs.

Possible explanations for these differing results include preanalytical or analytical factors, differences in laboratory settings, and potentially unrecognised environmental or clinical influences. Furthermore, both studies included relatively small numbers of patients, and additional research is needed to confirm these findings. It would also be of interest to investigate results from other countries, particularly those geographically more distant from Europe.

Overall, the clinical utility of measuring TgAA in dogs with newly diagnosed HA appears to be limited. We recommend using TgAA as an additional parameter alongside other thyroid hormone levels, clinical presentation and further diagnostic tests. Our data further demonstrate that complete resolution of AIT, including TgAA‐positive hypothyroidism, seems possible.

The major limitation of this study is the small sample size, which is due to the rarity of the disease. The comparison of thyroid parameters between groups A and B did not reveal significant differences; however, the limited sample size and statistical power may have resulted in a type II error, potentially masking true differences between the groups. Future studies with a larger case number, using a multicentre setting, are needed to confirm or refute these observations. Additionally, fine‐needle aspirates or biopsies were not obtained from the adrenal or thyroid glands of the dogs to detect lymphocytic inflammation, which could have confirmed an autoimmune reaction. These invasive procedures were avoided as the associated risks were considered to outweigh the potential benefits. Despite the absence of cytological or histopathological results, we presumed a diagnosis of AIT in cases where a positive TgAA titre was detected, and autoimmune HA based on the diagnostic tests performed and the exclusion of other potential causes (e.g., neoplasia and drug‐induced aetiologies). Additionally, not all dogs underwent follow‐up assessments for thyroid hormones and TgAA titres. There was variability in the duration of available rechecks for initially TgAA‐positive dogs, ranging up to a maximum of 1409 days. Furthermore, endogenous ACTH, fT4 concentrations and autoantibodies targeting TT4, T3 and TPO were not measured in any patient. The thyroid status of the included dogs was not confirmed via TSH stimulation test or scintigraphy due to the retrospective nature of the study and the limited availability of these diagnostic methods at our institution.

## CONCLUSION

In conclusion, the overall incidence of concurrent HA and hypothyroidism seems generally underestimated. Additional thyroid disease should be considered in dogs with HA exhibiting suitable clinical signs, and appropriate testing should be initiated. However, it is important to acknowledge the normalisation of AIT with cortisol replacement therapy in several patients diagnosed with HA. Delaying the initiation of thyroid hormone substitution and conducting regular follow‐up examinations are recommended to prevent the erroneous diagnosis of concurrent hypothyroidism. This approach will help avoid unnecessary lifelong thyroxine supplementation.

## AUTHOR CONTRIBUTIONS

*Conceptualisation, data curation, investigation, methodology, project administration, software, formal analysis, writing—original draft preparation (lead) and writing—reviewing and editing*: Christin Emming. *Data curation, investigation, methodology, project administration and software*: Sina Strey and Ina Leiter. *Conceptualisation, resources, project administration, writing—review and editing, and validation*: Reinhard Mischke and Holger Volk. *Conceptualisation, resources, project administration, writing—review and editing, and supervision*: Marion Schmicke and Johanna Rieder.

## CONFLICT OF INTEREST STATEMENT

The authors declare they have no conflicts of interest.

## FUNDING INFORMATION

The authors received no specific funding for the conduct or implementation of this study.

## ETHICS STATEMENT

This study was exempt from ethics committee approval as it involved a retrospective analysis of electronic data collected during clinician‐driven care provided to patients at the institution's hospital. No identifying information about clients or patients is included.

## Data Availability

The data that support the findings of this study are available from the corresponding author upon reasonable request.
